# B cells depletion as a value alternative therapeutic in pediatric autoimmune disorders: 3 case reports

**DOI:** 10.1186/1546-0096-9-S1-P69

**Published:** 2011-09-14

**Authors:** Joana Extreia, Inês Marques, Paula Afonso, Joaquim Rodrigues, Nuno Fernandes, Vera Silva

**Affiliations:** 1 Department of Pediatrics, Barreiro, Portugal; 2 Autoimmune Diseases Centre, Barreiro, Portugal

## Background

Approach to autoimmune diseases remains a challenge, not only by their clinical complexity but also because of frequent refractoriness to conventional treatment. We report 3 cases in which B cells depletion, with monoclonal antibody anti-CD20, appears as a therapeutic option to consider.

## Case 1

17-year-old boy referred to the Autoimmune Diseases Centre with symmetrical proximal muscle weakness, weight loss, dysphagia, periungual lesions and erythema of hands and eyelids with a year of evolution. Laboratory tests showed elevated lactate dehydrogenase, creatine kinase, transaminases and aldolase. Immunological/serological tests were negative. Capillaroscopy was compatible with Juvenile Dermatomyositis. Electromyography and muscle biopsy revealed nonspecific inflammatory myopathy. Corticosteroids were used but there was progression of disease. Rituximab was introduced with significant clinical improvement.

## Case 2

11-year-old girl diagnosed with Mixed Connective Tissue Disease for 1 year, with adverse reactions to corticosteroids. Completed 4 cycles of Rituximab with total regression of skin lesions and CD19 depletion. Eight months after, there was laboratory and clinical recurrence, with arthritis, vasculitis, weight loss, and elevation of CD19. The second cycle of Rituximab was performed uneventfully with clinical improvement and progressive CD19 normalization.

## Case 3

13-year-old girl diagnosed with Systemic Sclerosis with blood vessel, gastrointestinal and muscle/joint involvement. Despite the improvement of Raynaud's phenomenon with bosentan, she progressed to hip arthritis with gait impairment and severe pain. Refractory to conventional treatment, completed 4 cycles of Rituximab with clinical improvement, hip arthritis complete recovery and CD19 depletion.

## Conclusion

Rituximab seems to be an effective alternative for severe and refractory autoimmune diseases.

